# Chemotherapy combined with radiotherapy for successful treatment of angioimmunoblastic T-cell lymphoma: a case report

**DOI:** 10.1186/s13256-020-02489-4

**Published:** 2020-10-13

**Authors:** Yudi Xiong, Lei Yang, Jing Dai, Fuxiang Zhou, Yunfeng Zhou

**Affiliations:** 1 Hubei Cancer Clinical Study Center, Hubei Key Laboratory of Tumor Biological Behaviors, Zhongnan Hospital, Wuhan University, Wuhan, China; 2 Department of Radiation Oncology & Medical Oncology, Zhongnan Hospital, Wuhan University, 169 Donghu Road, Wuhan, 430071 China

**Keywords:** T-cell lymphoma, Angioimmunoblastic T-cell lymphoma, Chemotherapy, CHOP, TOMO radiotherapy

## Abstract

**Background:**

The incidence of angioimmunoblastic T-cell lymphoma is rare worldwide, and it has a poor prognosis. There is no proven or standard first-line therapy that works for the majority of patients with angioimmunoblastic T-cell lymphoma because of the rarity of this disease. The treatment and management are challenging for clinicians.

**Case presentation:**

This report presents the diagnosis and treatment of a 65-year-old Chinese man who presented with cough and lymph node swellings in the left axillary region. The patient was diagnosed with angioimmunoblastic T-cell lymphoma. He underwent eight cycles of chemotherapy with CHOP (cyclophosphamide, hydroxydaunorubicin, oncovin, prednisone) followed by TOMO radiotherapy (helical tomotherapy, a kind of radiotherapy for cancer treatment using spiral computed tomographic scanning). After treatment, the therapeutic effects were evaluated by magnetic resonance imaging and computed tomography about every 3 months. The patient recovered well with no sign of tumor recurrence and no obvious severe treatment-related adverse effects.

**Conclusion:**

This treatment experience indicates an essential role for the combination of radiation therapy with CHOP, which may have a better prognosis than treatments without radiation therapy. But challenges warrant further validation in prospective studies.

## Background

Angioimmunoblastic T-cell lymphoma (AITL) is a rare form of non-Hodgkin lymphoma that accounts for 1–2% of all people with non-Hodgkin lymphoma [1]. However, it is one of the most common types of peripheral T-cell lymphoma [2, 3]. It was first reported clinically in 1974 and characterized as a very aggressive disease associated with rash, pleural effusions, polyclonal hypergammaglobulinemia, ascites, and autoimmune phenomenon [4]. It has a poor prognosis, with 5-year progression-free survival (PFS) being approximately 25% [5]. A review found only one of seven patients could survive longer than 3 years [6].

So far, because of the rarity of this disease, there is no proven or standard first-line therapy that works for the majority of patients with AITL. The patients are treated with a traditional non-Hodgkin lymphoma therapy or in clinical trials [7]. Here, in order to provide some information on its treatment, we report a case of a patient with AITL who was treated successfully with CHOP (cyclophosphamide, hydroxydaunorubicin, oncovin, prednisone) in combination with tomotherapy.

## Case presentation

**Fig. 1:**
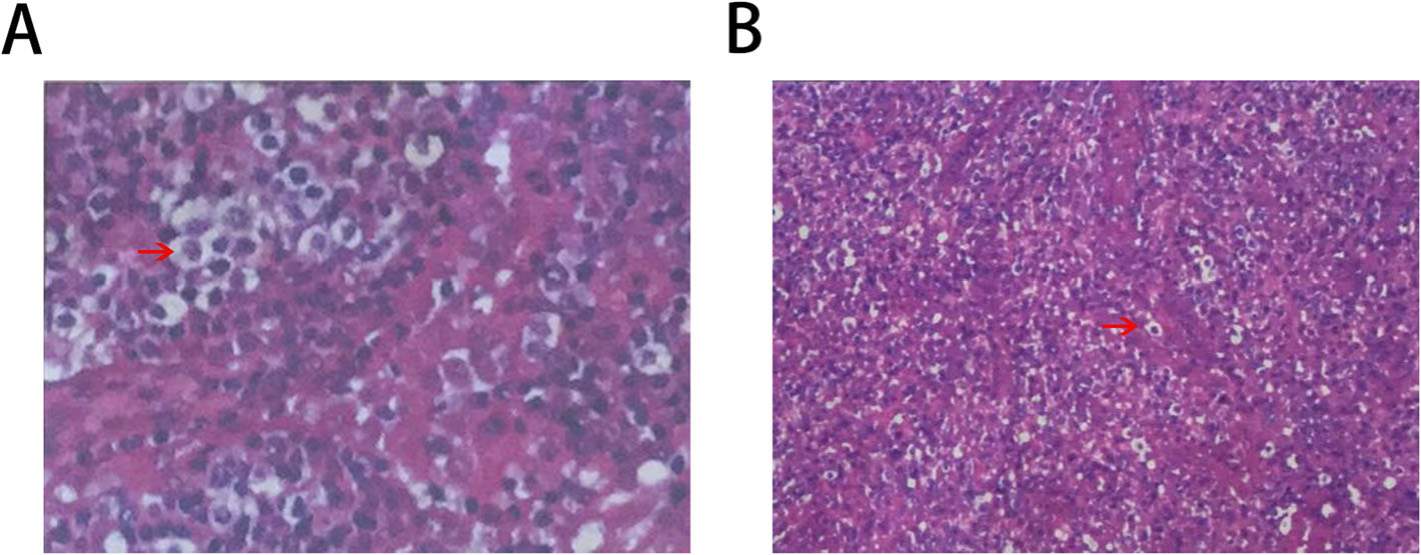
Tissue features of the patient. **a** The result of Hematoxylin-eosin staining of tissues performed by the local hospital, **b** The result of Hematoxylin-eosin staining of tissues performed by our hospital. There are small to medium-sized lymphocytes (red arrow), transparent cytoplasm, and vascular hyperplasia in lymph nodes (**a** 400X; **b** 200X). Combined with the results of immunohistochemistry, the two hospitals diagnosed it as angioimmunoblastic T-cell lymphoma (AITL)

A 65-year-old Chinese man was admitted to the local hospital on December 28, 2017, with a 1-month history of cough and lymph node swellings that had grown gradually. On the basis of a left-side axillary lymph node biopsy specimen, he was diagnosed with AITL (Fig. 1a), and then the patient came to our department for treatment. We performed a detailed physical examination but only found bilateral cervical multiple lymphadenopathy without fever, cough, chest tightness, and edema. His laboratory test abnormal findings were as follows: red blood cell count, 3.76 × 10^12^/L; hemoglobin, 7.5 g/dl; aspartate transaminase/alanine transaminase ratio, 2.12 (normal range 0.2 to 2.0); albumin, 26.6 g/L; prothrombin time, 12.8 seconds; prothrombin normalized ratio, 1.17; D-dimer stock solution, 1989 ng/ml (normal range, 0 to 500 ng/ml). A bone marrow aspiration smear showed no abnormal cells.

**Fig. 2:**
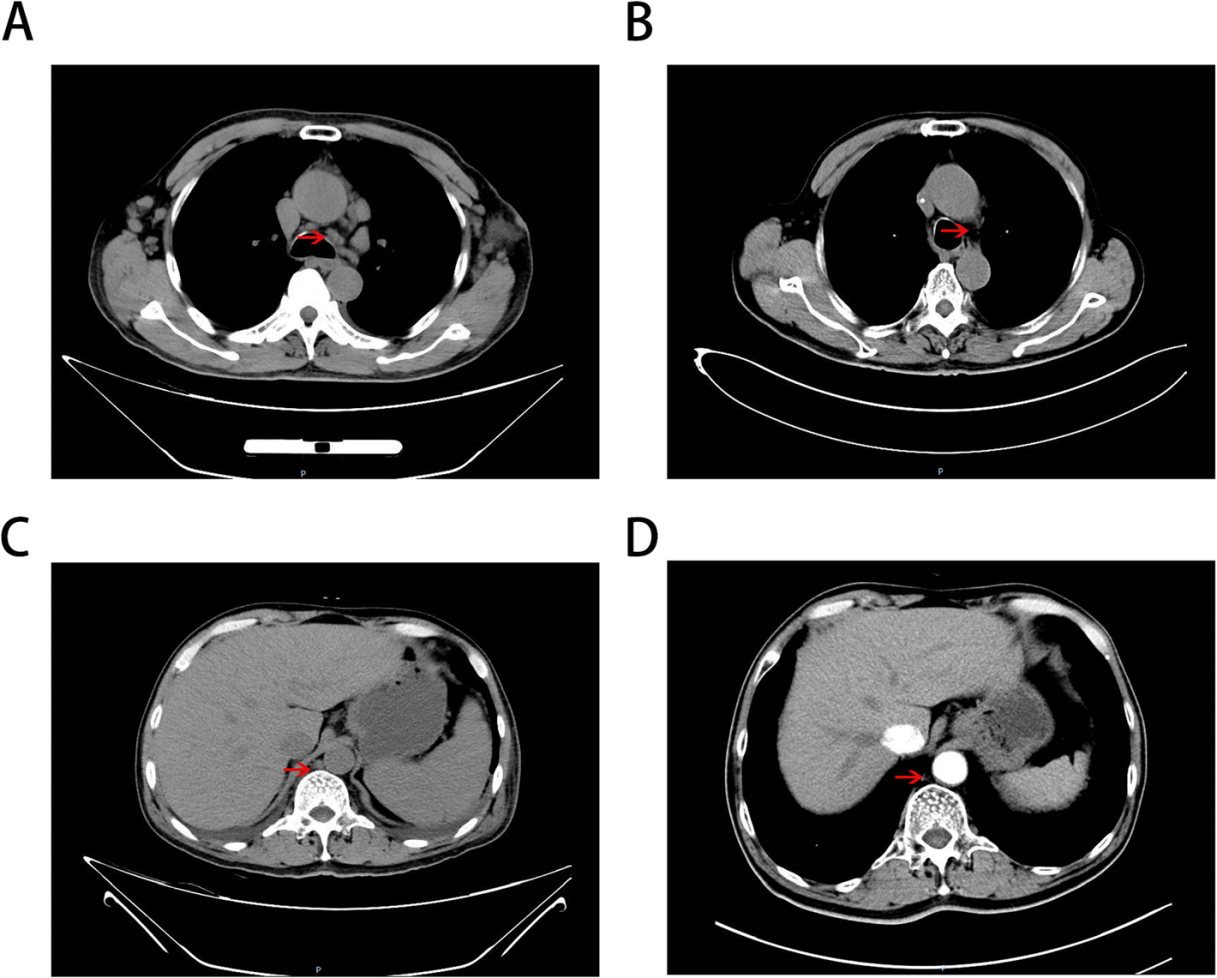
The chest and abdominal computed tomography (CT) **a** Revealed bilateral axillary and mediastinal lymphadenopathy (arrow), one of which was about 13 × 18mm in the mediastinum before treatment. **b** Showed lymph node was significantly shrink after treatment (arrow). **c** Revealed para-aortic and para-splenic lymphadenopathy (arrow), one of which was about 16mm before treatment. **d** Revealed no obvious lymphadenopathy after treatment (arrow)

The patient’s cervical enhanced magnetic resonance imaging (MRI) examination revealed bilateral cervical multiple lymphadenopathy, one of which was about 22 × 18 mm. Computed tomography (CT) of his chest revealed bilateral axillary and mediastinal lymphadenopathy, one of which was about 13 × 18 mm in the mediastinum; nodules in the upper and middle lobes of the right lung; pneumonitis of bilateral lower lobes; bilateral pleural effusion; and bilateral pleural thickening (Fig. 2a). Abdominal enhanced CT revealed para-aortic and parasplenic lymphadenopathy, one of which was about 16 mm (Fig. 2c). Pelvic CT revealed pelvic and bilateral groin lymphadenopathy, one of which was about 13 × 10 mm. Histopathological study was performed again and indicated AITL, which showed small to medium-sized lymphocytes, transparent cytoplasm, and vascular hyperplasia in the lymph nodes (Fig. 1b). The results of immunohistochemistry were as follows: CD34^+^, CD4^+^, CD38^+^, Bcl-6^+^, CD21^+^, CD20^+^, CD23^+^, CD5^+^, CD10^−^, PD-1^+^, and Ki-67 (50%). On the basis of these findings, a diagnosis of AITL was made, and the staging of lymphoma was IIIA. The patient underwent eight cycles of chemotherapy with CHOP from January 12, 2018, to June 21, 2018, and grade I leukopenia occurred during chemotherapy. No other obvious severe treatment-related toxicities were noted. To evaluate the therapeutic effects, positron emission tomography was performed after six cycles of chemotherapy, which showed that there was still hypermetabolic lymphadenopathy in the cervical region. We were considering whether radiotherapy should be started after six chemotherapy cycles or after eight cycles. After discussing this with our team, we planned TOMO radiotherapy (planning target volume–clinical target volume 40 Gy/20 fractions) to the head and neck lymphatic drainage area after completing the two remaining chemotherapy cycles. The patient only underwent 18 fractions from August 2, 2018, because of poor tolerance.

Two months after treatment, the patient’s leukopenia normalized. His cervical enhanced MRI examination revealed bilateral cervical lymphadenopathy, with the bigger one being about 9 × 6 mm. His chest CT scan revealed bilateral axillary and mediastinal small lymph nodes. His abdominal enhanced CT and pelvic enhanced CT revealed that his para-aortic and parasplenic lymphadenopathy was significantly reduced. Eight months later, on April 22, 2019, the patient was reexamined in our hospital. His cervical enhanced MRI examination revealed bilateral cervical small lymph nodes. His chest enhanced CT scan revealed several mediastinal small lymph nodes (Fig. 2b). His abdominal enhanced CT and pelvic CT revealed no obvious lymphadenopathy (Fig. 2d). The patient was reexamined in our hospital on June 18, 2020. On the basis of MRI and CT findings, no recurrence of lymphadenopathy was recognized, and laboratory examinations also found no obvious abnormal indicators. The remnant of the tumor continued to regress, and the patient did not develop obvious severe treatment-induced toxicities.

## Discussion

AITL is a rare form of non-Hodgkin lymphoma that is characterized as a very aggressive disease associated with rash, pleural effusions, polyclonal hypergammaglobulinemia, ascites, and an autoimmune phenomenon. It has a poor prognosis, with 5-year PFS around 25%, and only one of seven patients in one study survived longer than 3 years [6]. The diagnosis and therapeutic management of AITL may require the coordinated efforts of a team of medical professionals, such as medical oncologists, hematologists, radiation oncologists, oncology nurses, surgeons, dietitians, and/or other healthcare professionals. The pathological features of AITL are diffuse effacement and neovascular infiltration by plasma cells and immunoblasts and vascular proliferation. The expression of CD3, CD10, CD28, CD30, CXCL13, ICOS, and PD1 can be detected with histopathological staining of AITL cells. Therapies used to treat individuals with AITL include corticosteroids, watching and waiting, single-agent chemotherapy, and multiagent chemotherapy [7]. Owing to lack of standard first-line therapy, there are several available treatments, and many different types of drugs have been used or are being studied to treat individuals with AITL, such as chemotherapy (CHOP/CHOEP/COEP-B [8, 9], DHAP/ESHAP combination regimens [10]), targeted therapy (alemtuzumab [11], bortezomib [12]), and autologous stem cell transplant [13, 14]. Although some agents have been used, the results have not been satisfactory (Table 1). The median PFS of AITL maybe shorter than reported in Table 1. The PFS of our patient is approximately 31 months, however, which is longer than in prior studies including CHOP. This suggests that CHOP combined with radiotherapy may be better than CHOP alone to treat these patients.

**Table 1:** Agents used in treatment of angioimmunoblastic T-cell lymphoma

Agent [reference]	AITL (no.)	ORR/CR (%)	Median PFS (months)
Romidepsin [15]	27	30/19	4
Pralatrexate [16]	13	8/NR	3.5
Bendamustine [17]	32	NR	3.6
Brentuximab vedotin [18]	13	54/38	2.6
Lenalidomide [19]	7	29/0	3.2
CHOP [20]	33	NR/61	26

*Abbreviations: AITL* Angioimmunoblastic T-cell lymphoma, *CR* Complete response, *ORR* Overall response rate, *PFS* Progression-free survival

Few clinical trials have involved radiotherapy in the therapeutic management of AITL. Clinical oncologists tend to find drugs against the tumor, with numerous new systemic therapies available for treatment of lymphoma. Currently, the clinical research on AITL is either drug therapy or stem cell transplant therapy. The use of radiation therapy should not be overlooked or should at least be considered, even though there are individual differences and different therapies in these cases. Radiation therapy has been an integral component of lymphoma treatment for decades, which has been described as the most effective single agent in the treatment of lymphoma [21]. We chose TOMO radiotherapy to treat our patient, because TOMO increases the target area irradiation dose, thereby improving the cure rate and the survival rate and reducing the incidence of complications. Therefore, it has obvious advantages over conventional radiotherapy in terms of overall treatment cost-effectiveness. For comparison with recorded data, we have a proper follow-up in order to evaluate the therapeutic effects after treatment. We told the patient to return to our hospital for reexamination every 3 months for the first 2 years, every 6 months in years 3–5 , and once per year after 5 years. The patient has good treatment compliance. He came to be reexamined every 3–4 months. In addition, as of July 28, 2020, he is still alive with the remnant of the tumor continuing to regress.

## Conclusion

We report a case of a 65-year-old man with AITL who underwent eight cycles of chemotherapy with CHOP followed by TOMO radiotherapy. The treatment was well tolerated. To date, the patient has recovered well with no sign of tumor recurrence and no obvious severe treatment-related adverse effects. Therefore, radiation therapy followed by chemotherapy with CHOP might be an effective strategy for AITL and should not be ignored during these patients’ treatments, which warrants further validation in prospective studies.

## Data Availability

The datasets used and analyzed during the current study are available from the corresponding author on reasonable request.

## Abbreviations

AITL: Angioimmunoblastic T-cell lymphoma
CHOP: Cyclophosphamide, hydroxydaunorubicin, oncovin, prednisone
CR: Complete response
CT: Computed tomography
MRI: Magnetic resonance imaging
ORR: Overall response rate
PFS: Progression-free survival

## References

[1] Non-Hodgkin’s Lymphoma Classification Project. A clinical evaluation of the International Lymphoma Study Group classification of non-Hodgkin’s lymphoma. Blood. 1997;89:3909–18. 9166827

[2] de Leval L, Gisselbrecht C, Gaulard P. Advances in the understanding and management of angioimmunoblastic T-cell lymphoma. Br J Haematol. 2010;148:673–89. 10.1111/j.1365-2141.2009.08003.x 19961485

[3] de Leval L, Parrens M, Le Bras F, Jais JP, Fataccioli V, Martin A, *et al.* Angioimmunoblastic T-cell lymphoma is the most common T-cell lymphoma in two distinct French information data sets. Haematologica. 2015;100:E361–4. 10.3324/haematol.2015.126300 PMC4800690 26045291

[4] Frizzera G, Moran EM, Rappaport H. Angio-immunoblastic lymphadenopathy with dysproteinaemia. Lancet. 1974;1:1070–3. 10.1016/s0140-6736(74)90553-4 4135245

[5] Kameoka Y, Takahashi N, Itou S, Kume M, Noji H, Kato Y, *et al.* Analysis of clinical characteristics and prognostic factors for angioimmunoblastic T-cell lymphoma. Int J Hematol. 2015;101:536–42. 10.1007/s12185-015-1763-7 25739382

[6] Reiser M, Josting A, Soltani M, Staib P, Salzberger B, Diehl V, *et al.* T-cell non-Hodgkin’s lymphoma in adults: clinicopathological characteristics, response to treatment and prognostic factors. Leuk Lymphoma. 2002;43:805–11. 10.1080/10428190290016926 12153168

[7] Ma H, Abdul-Hay M. T-cell lymphomas, a challenging disease: types, treatments, and future. Int J Clin Oncol. 2017;22:18–51. 10.1007/s10147-016-1045-2 PMC7102240 27743148

[8] Trumper L, Zwick C, Ziepert M, Hohloch K, Schmits R, Mohren M, *et al.* Dose-escalated CHOEP for the treatment of young patients with aggressive non-Hodgkin’s lymphoma: I. A randomized dose escalation and feasibility study with bi- and tri-weekly regimens. Ann Oncol. 2008;19:538–44. 10.1093/annonc/mdm497 18212092

[9] Sung HJ, Kim SJ, Seo HY, Sul HR, Choi JG, Choi IK, *et al.* Prospective analysis of treatment outcome and prognostic factors in patients with T-cell lymphomas treated by CEOP-B: single institutional study. Br J Haematol. 2006;134:45–53. 10.1111/j.1365-2141.2006.06124.x 16803566

[10] Yao YY, Tang Y, Zhu Q, Zhuang Y, Cheng YM, Wang L, *et al.* Gemcitabine, oxaliplatin and dexamethasone as salvage treatment for elderly patients with refractory and relapsed peripheral T-cell lymphoma. Leuk Lymphoma. 2013;54:1194–200. 10.3109/10428194.2012.739286 23061678

[11] Binder C, Ziepert M, Pfreundschuh M, Duhrsen U, Eimermacher H, Aldaoud A, *et al.* CHO(E)P-14 followed by alemtuzumab consolidation in untreated peripheral T cell lymphomas: final analysis of a prospective phase II trial. Ann Hematol. 2013;92:1521–8. 10.1007/s00277-013-1880-4 PMC3790248 23978945

[12] Kim SJ, Yoon DH, Kang HJ, Kim JS, Park SK, Kim HJ, *et al.* Bortezomib in combination with CHOP as first-line treatment for patients with stage III/IV peripheral T-cell lymphomas: a multicentre, single-arm, phase 2 trial. Eur J Cancer. 2012;48:3223–31. 10.1016/j.ejca.2012.06.003 22770877

[13] d’Amore F, Relander T, Lauritzsen GF, Jantunen E, Hagberg H, Anderson H, *et al.* Up-front autologous stem-cell transplantation in peripheral T-cell lymphoma: NLG-T-01. J Clin Oncol. 2012;30:3093–9. 10.1200/JCO.2011.40.2719 22851556

[14] Mehta N, Maragulia JC, Moskowitz A, Hamlin PA, Lunning MA, Moskowitz CH, *et al.* A retrospective analysis of peripheral T-cell lymphoma treated with the intention to transplant in the first remission. Clin Lymphoma Myeloma Leuk. 2013;13:664–70. 10.1016/j.clml.2013.07.005 24035712

[15] Coiffier B, Pro B, Prince HM, Foss F, Sokol L, Greenwood M, *et al.* Results from a pivotal, open-label, phase II study of romidepsin in relapsed or refractory peripheral T-cell lymphoma after prior systemic therapy. J Clin Oncol. 2012;30:631–6. 10.1200/JCO.2011.37.4223 22271479

[16] O’Connor OA, Pro B, Pinter-Brown L, Bartlett N, Popplewell L, Coiffier B, *et al.* Pralatrexate in patients with relapsed or refractory peripheral T-cell lymphoma: results from the pivotal PROPEL study. J Clin Oncol. 2011;29:1182–9. 10.1200/JCO.2010.29.9024 PMC3083873 21245435

[17] Damaj G, Gressin R, Bouabdallah K, Cartron G, Choufi B, Gyan E, *et al.* Results from a prospective, open-label, phase II trial of bendamustine in refractory or relapsed T-cell lymphomas: the BENTLY trial. J Clin Oncol. 2013;31:104–10. 10.1200/JCO.2012.43.7285 23109692

[18] Horwitz SM, Advani RH, Bartlett NL, Jacobsen ED, Sharman JP, O’Connor OA, *et al.* Objective responses in relapsed T-cell lymphomas with single-agent brentuximab vedotin. Blood. 2014;123:3095–100. 10.1182/blood-2013-12-542142 PMC4425442 24652992

[19] Dueck G, Chua N, Prasad A, Finch D, Stewart D, White D, *et al.* Interim report of a phase 2 clinical trial of lenalidomide for T-cell non-Hodgkin lymphoma. Cancer. 2010;116:4541–8. 10.1002/cncr.25377 20572046

[20] Pautier P, Devidas A, Delmer A, Dombret H, Sutton L, Zini JM, *et al.* Angioimmunoblastic-like T-cell non Hodgkin’s lymphoma: outcome after chemotherapy in 33 patients and review of the literature. Leuk Lymphoma. 1999;32:545–52. 10.3109/10428199909058412 10048427

[21] Yahalom J. Indications to radiotherapy for lymphoma in 2016: what is standard of care and what remains controversial? [abstract]. Radiother Oncol. 2016;119 Suppl 1:S236.

